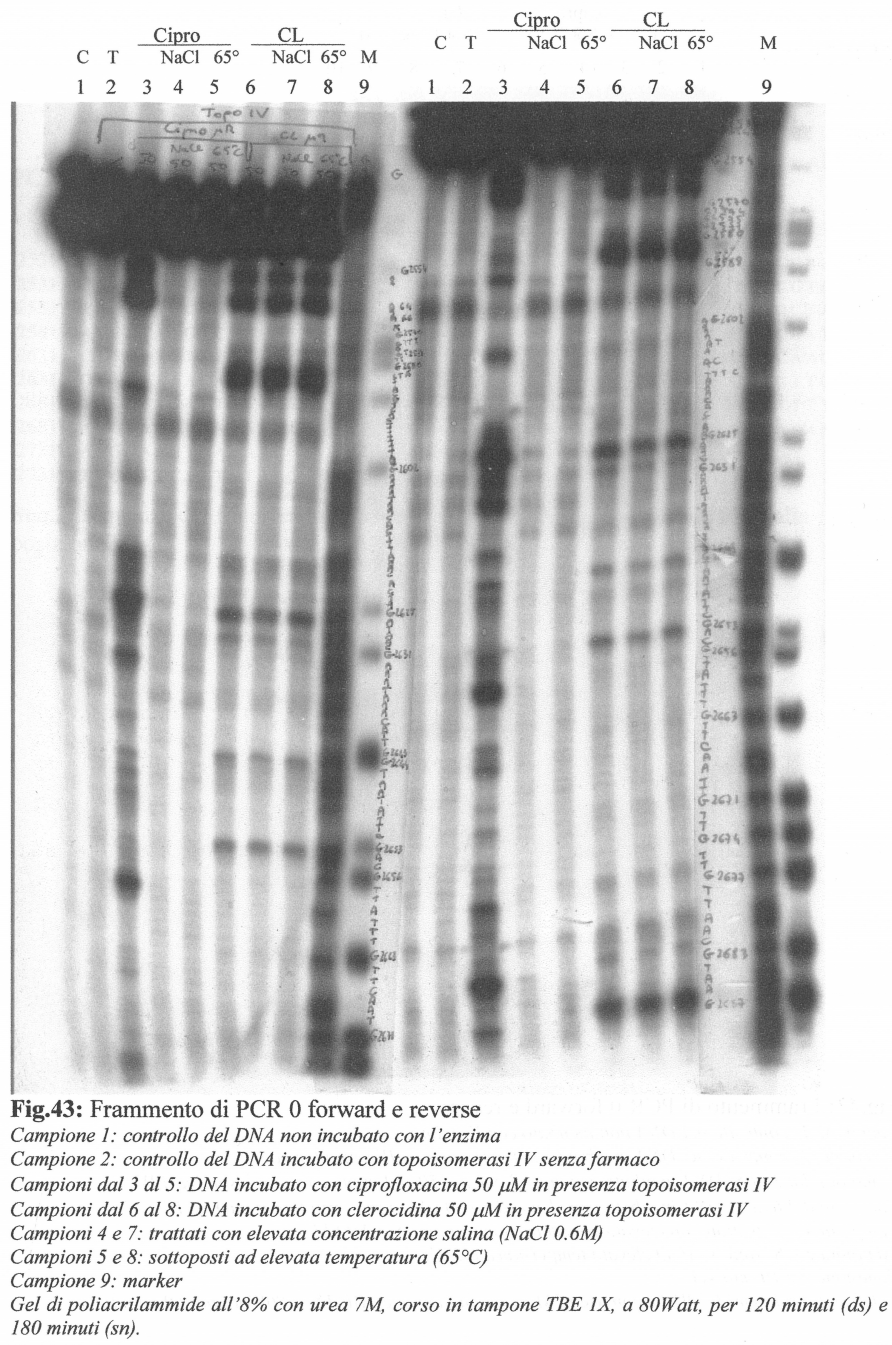# Correction to ‘Clerocidin interacts with the cleavage complex of *Streptococcus pneumoniae* topoisomerase IV to induce selective irreversible DNA damage’

**DOI:** 10.1093/nar/gkac1149

**Published:** 2022-11-23

*Nucleic Acids Research*, Volume 34, Issue 7, 1 April 2006, Pages 1982–1991, https://doi.org/10.1093/nar/gkl127

The authors regret the accidental duplication of the first two lanes in Figure 4 of their article.

While the authors do not have access to the original raw data which was generated over 16 years ago, the same experimental data in its earliest form was included in the Master's thesis of one of the authors, Giulia Giaretta, ‘Confronto dell’attività di clerocidina e ciprofloxacin su topoisomerase procariotiche da Gram’ + e Gram -" submitted to and awarded by the University of Padua, Italy, in 2003. A copy of the image is provided below. The experiment on the right corresponds to Figure 4 in the NAR article.

The figure comes from an X-ray film where the author has stuck a transparent tape close to the marker lane to write the DNA sequence. The sequence corresponding to the experiment on the left overlaps lane 1 of the experiment on the right which later became Figure 4 in the NAR article.

This correction does not affect the results or conclusions of the article.

These details have been corrected only in this correction notice to preserve the published version of record.